# Efficacy and safety evaluation of a novel trioxaquine in the management of cerebral malaria in a mouse model

**DOI:** 10.1186/s12936-017-1917-6

**Published:** 2017-07-03

**Authors:** Onyango C. Odhiambo, Hannah N. Wamakima, Gabriel N. Magoma, Peter G. Kirira, Bonface J. Malala, Francis T. Kimani, Francis W. Muregi

**Affiliations:** 1 Department of Biochemistry, Jomo Kenyatta University of Agriculture and Technology, P.O. Box 62000, Nairobi, Kenya; 2 Department of Pharmaceutical Sciences, Mount Kenya University, P.O. Box 342-01000, Thika, Kenya; 3 Department of Physical Sciences, Mount Kenya University, P.O. Box 342-01000, Thika, Kenya; 4 Department of Biological Sciences, Mount Kenya University, P.O. Box 342-01000, Thika, Kenya; 5 Centre for Biotechnology Research and Development, Kenya Medical Research Institute (KEMRI), P.O. Box 54840, Nairobi, Kenya

**Keywords:** Covalent bitherapy, Polypharmacology, Trioxaquine, Cerebral malaria

## Abstract

**Background:**

The emergence of multidrug-resistant strains of *Plasmodium falciparum* poses a great threat of increased fatalities in cases of cerebral and other forms of severe malaria infections in which parenteral artesunate monotherapy is the current drug of choice. The study aimed to investigate in a mouse model of human cerebral malaria whether a trioxaquine chemically synthesized by covalent linking of a 4,7-dichloroquinoline pharmacophore to artesunate through a recent drug development approach termed ‘covalent bitherapy’ could improve the curative outcomes in cerebral malaria infections.

**Methods:**

Human cerebral malaria rodent model, the C57BL/6 male mice were infected intraperitoneally (ip) with *Plasmodium berghei* ANKA and intravenously (iv) treated with the trioxaquine from day 8 post-infection (pi) at 12.5 and 25 mg/kg, respectively, twice a day for 3 days. Treatments with the trioxaquine precursors (artesunate and 4,7-dichloroquine), and quinine were also included as controls. In vivo safety evaluation for the trioxaquine was done according to Organization for Economic Co-operation and Development (OECD) guidelines 423, where female Swiss albino mice were orally administered with either 300 or 2000 mg/kg of the trioxaquine and monitored for signs of severity, and or mortality for 14 days post-treatment.

**Results:**

The trioxaquine showed a potent and a rapid antiplasmodial activity with 80% parasite clearance in the first 24 h for the two dosages used. Long-term parasitaemia monitoring showed a total parasite clearance as the treated mice survived beyond 60 days post-treatment, with no recrudescence observed. Artesunate treated mice showed recrudescence 8 days post-treatment, with all mice in this group succumbing to the infection. Also, 4,7-dichloroquinoline and quinine did not show any significant parasitaemia suppression in the first 24 h post-treatment, with the animals succumbing to the infection.

**Conclusion:**

Covalent bitherapy proves to be a viable source of urgently needed new anti-malarials for management of cerebral malaria, and this polypharmacology approach could be a potential strategy to protect artesunate from parasite resistance and in potentially improving clinical outcomes in severe forms of malaria infections.

## Background

Despite many years of research and great progress in the fight against malaria [1], cerebral malaria (CM) has remained the most dreadful severe complication of *Plasmodium falciparum,* with a higher burden greatly felt in sub-Saharan Africa [2], a continent that already battles the effect of other diseases such as tuberculosis and HIV/AIDS [3]. Children 5 years old and younger are the most susceptible, with a staggering 90% CM related fatalities [4]. With no effective vaccine against malaria yet, chemotherapy has remained the mainstay of malaria management [5]. Extremely limited options for chemotherapy in CM have been a great challenge [5]. For decades, quinine has been the drug of choice for management of CM [6]. Concerns over increasing resistance to the quinoline-based drugs such as chloroquine (CQ) prompted World Health Organization (WHO) policy change to use of artemisinin-based drugs, such as artemether and artesunate. In management of CM, preference has been given to parenteral artesunate due to its superior efficacy and survival outcome in comparison to intramuscular artemether [7, 8]. Despite effective CM chemotherapy with artesunate, mortality rate still remains as high as 15–25% among treated children while those that survive the acute episodes of CM suffer long-term neurological sequelae including epilepsy [9].

*Plasmodium falciparum,* the most lethal pathogen among the *Plasmodium* species causing up to 90% of all malaria-related deaths in sub-Saharan Africa has shown multi-drug resistance [10]. Reports of parasite resistance against the WHO-recommended artemisinin (ART) derivatives—the first-line anti-malarials—have already been documented, prompting an urgent need for new drugs [10]. Extremely low success rates for new chemotherapy in the development pipeline to proceed to clinical trials [11] has necessitated a rational deployment of the available effective drugs to optimize their useful therapeutic life and also to protect them from being rendered ineffective due to parasite resistance [12]. Currently, ART derivatives are used in combination with longer durational anti-malarials in the artemisinin-based combination therapy (ACT) strategy, in order to protect them against resistance [5]. Unfortunately, in CM, artesunate has often been used as a monotherapy [7], thus calling for a polypharmacology strategy in such a scenario, especially in sub-Saharan Africa.

In the long and difficult search to develop effective, cheaper and urgently needed anti-malarial drugs, many research groups have employed the emerging strategy in medicinal chemistry called hybridization or covalent bitherapy [12–14]. This is a rational drug design approach based on covalent linking of drug pharmacophores into a single hybrid molecule, termed as hybrid drug, dual drug or conjugates [12]. The resulting hybrid drugs have been reported to possess superior efficacy to either the individual precursors alone or their co-formulations [15]. This approach reduces the risk of treatment failure and also offers resistance protection to the partner drug or to both drugs [16], and is believed to be cheaper since only the active moieties are covalently linked, thus avoiding the long, laborious and expensive drug development procedures of individual drugs before co-formulation [16]. Trioxaquines, which are hybrid drugs arising from conjugation of 1,2,4-endoperoxide bridge of the artemisinins and a quinoline pharmacophore, are demonstrative of the validity of this approach [12].

**Fig. 1:**
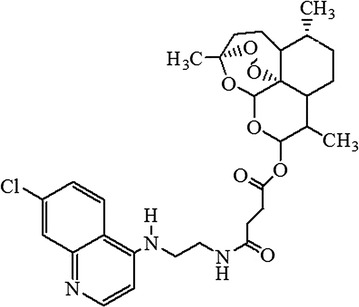
Structure of trioxaquine: *N*-(7-chloroquinolin-4-ylamino)-ethyl-artesunate-19-carboxamide

In this study, a trioxaquine termed *N*-(7-chloroquinolin-4-ylamino)-ethyl-artesunate-19-carboxamide (Fig. 1) was evaluated for curative effect of CM in mouse model. The dual drug was previously synthesized and demonstrated to possess remarkable antiplasmodial effect against blood stage rodent malaria parasite (ED_50_ and ED_90_ of 5.5 and 13.5 mg/kg, respectively) [17]. The dual drug had also shown a remarkable in vitro antiplasmodial activity against CQ-sensitive (D6) (IC_50_, 6.89 ng/mL) and CQ-resistant (W2) (IC_50_, 3.62 ng/mL) *P. ​falciparum* isolates [17].

## Methods

### Parasites, hosts and drugs

*Plasmodium berghei* (ANKA strain) was used in the study. This parasite, maintained at −80 °C in Kenya Medical Research Institute (KEMRI) at the Centre for Biotechnology, Research, and Development (CBRD), was thawed and revived by intraperitoneal (ip) inoculation into male Swiss albino mice. The mice served as parasite donor for passaging into C57BL/6 male mice weighing 25 ± 2 g, used as a mouse model of CM. The mice were subsequently randomized into groups of five per cage and fed with pellets and water, ad libitum [18].

The test drugs, namely artesunate, 4,7-dichloroquinoline and quinine were purchased from Sigma Aldrich^®^, while the trioxaquine, a kind gift from Prof. Francis W Muregi, was previously synthesized by linking the 4,7-dichloroquinoline to a linker and coupling the product to artesunate [17]. On the day of administration, artesunate, the dichloroquinoline and the trioxaquine were dissolved in a solution of 70% Tween-80 (d = 1.08 g/ml) and 30% ethanol (d = 0.81 g/ml) at a final diluted concentration of 7% (v/v), and 3% (v/v) for Tween 80 and ethanol, respectively [18]. Quinine was dissolved in normal saline.

### Infection of experimental mice

The donor mice were anaesthetized and the infected red blood cells collected using heparinized syringe via cardiac puncture. The parasitaemia was then adjusted downwards using sterilized phosphate-buffered saline (PBS). Male C57BL/6 mice were then inoculated ip with approximately 1 × 10^5^ parasitized erythrocytes (pRBCs) at inoculum of 0.2 ml. The infected mice were then randomized in plastic cages in groups of five and observed daily for signs of experimental cerebral malaria (ECM), including rough coat, limb paralysis, ataxia, convulsions, and or coma [19, 20]. In C57BL/6, ECM signs usually occur between day 6 and 9 post-infection (pi) [19, 20]. Giemsa-stained thin blood smear slides were also observed under light microscope (×1000) to establish infection.

### In vivo evaluation of the trioxaquine in cerebral malaria rodent models

The treatment of CM in experimental mice with established infection was carried out by the approach of Ryley and Peters [21] in which treatment with a drug was initiated when the infection was already well established and the mice had exhibited clear signs for cerebral malaria. Based on the previous results of the 4-day suppressive test that had been used to evaluate chemosuppression ability of the trioxaquine in vivo [17], a dosage of 12.5 and 25 mg/kg were administered intravenously (iv) twice daily for 3 days through the lateral tail veins from day 8 pi. Three positive control groups were also similarly treated with artesunate (12.5 mg/kg), quinine (60 mg/kg), and 4,7-dichloroquinoline (12.5 mg/kg). A negative control group that received only the drug vehicle was also included. Parasitaemia of each mouse was determined by microscopic examination of Giemsa-stained, thin blood smears prepared from mouse tail blood. Slides were coded and infected red blood cell (iRBC) count was done microscopically in at least five microscopic fields, each with an approximation of 200–400 iRBCs. Smears were done at 24 h post-treatment, and 48 h post-treatment later to monitor progression of parasitaemia. Parasitaemia counts of blood films from each mouse were processed using Microsoft^®^ Excel (Microsoft Corp.). Percentage (%) parasite suppression (chemosuppression) at each drug dose was determined as described by Muregi et al. [22]:

$${\text{Percentage suppression}} = 100 - \left( {[{\text{mean parasitaemia treated}} \div {\text{mean parasitaemia untreated}}] \times 100} \right).$$

The survival of mice was monitored for up to 60 days after the end of the 3-day treatment. All RBC counts and parasitaemia levels were expressed as mean values ± standard deviations and the parasitaemia data were processed using the one-way analysis of variance (ANOVA). Mean parasitaemia between each experimental group relative to controls was compared using Student’s t test with a probability of 5% (*p* < 0.05) considered significant.

### Evaluation of the integrity of mouse blood–brain barrier

C57BL/6 mice were injected iv with 200 μl of 2% (w/v) Evans Blue (Fischer Scientific^®^) dye on day 8 pi after the experimental animals had exhibited clear signs for cerebral malaria. The animals were euthanized after 1 h for brain extraction to assess the extent of brain staining with the dye and the images documented. The principle behind this method is based upon the formation of bonds between the dye and plasmatic albumin and, in the event of brain blood vessels leakage due to compromised blood–brain barrier (BBB), the dye–protein complex migrates to the tissue, impregnating it in a tone of blue visible to the naked eye [23].

### Safety evaluation of the trioxaquine

To evaluate the safety of the trioxaquine, the OECD Test Guidelines 423 (Acute Toxic Class Method, adopted on 17 December 2001) were adopted. This is a protocol that makes use of three uninfected animals of same gender, especially females, at every dosage category and assessment based on death and or signs of severity observed with the dosed animals. Averagely, two to four dosage steps in the range of 5, 50, 300 and 2000 mg/kg are considered enough in making judgement on the acute oral toxicity of the test compound [24].

Two fixed dose levels of 300 and 2000 mg/kg were used and dosing was done in a stepwise manner starting with the lowest dose upwards. Swiss albino female mice weighing 20 ± 2 g were used where the animals were first fasted for 4 h, after which they were dosed orally with the above dosages. The start, duration and severity of toxic signs of the test compound, such as changes in skin and fur, eyes, mucous membranes, tremors, convulsions, salivation, diarrhoea, coma, and even death were monitored. The animals were observed individually at least once during the first 30 min, periodically during the first 24 h with special attention given during the first 4 h, and daily thereafter for the next 14 days.

### Ethical statement

The use of mice in this research work was approved by KEMRI's Animal Care and Use Committee (ACUC) (Approval No: KEMRI/ACUC/01.04.16) and the Scientific Ethical Review Unit (SERU) (Permit No: KEMRI/RES/7/3/1/2016). All mice that were deemed to have completed their intended role in the study were euthanized using 150 mg/kg body weight sodium pentobarbital solution injected ip, placed in biohazard bags and incinerated.

### Data analysis

Data analysis was performed using SPSS version 16. All values are expressed in means (±SDs). Cumulative survival rates were calculated according to Kaplan–Meier method and groups were compared using the log-rank test. Parasitaemia courses were compared by analysis of variance (ANOVA). Parasitaemia before and at 24 h after treatment were compared by paired Student t test. *P* value <0.05 was considered to be statistically significant.

## Results

### Assessment of the therapeutic effect of the trioxaquine

Treatment was initiated on day 8 pi after the experimental animals were clearly diagnosed with ECM or having presented with at least one or both well-recognized signs of ECM in animal models, including neurological perturbations such as ataxia, rough coat, limb paralysis, *inter alia*. Parasitaemia load reduction for each test drug within the first 24 h post-treatment is presented in Table 1. The parasitaemia was monitored for 60 days post-treatment.

**Table 1:** Parasite load reduction (percentage) of each drug within 24 h after the first day of treatment with the trioxaquine, artesunate, 4,7-dichloroquinoline and quinine against *Plasmodium berghei* ANKA in C57BL/6 mice using the established infection test

Drug/dosage (mg/kg)	Day 8 pi mean parasitaemia ± SD	24 h post-treatment parasitaemia ± SD	48 h post-treatment parasitaemia ± SD	Percentage parasitaemia clearance in 24 h	Recrudescence
Trioxaquine					
25	11.73 ± 0.06	0.42 ± 0.11	0.00	96.4	Not observed
12.5	11.69 ± 0.03	1.56 ± 0.08	0.00	86.6	Not observed
Artesunate					
12.5	11.75 ± 0.05	1.77 ± 0.04	0.00	84.8	Observed
4,7-Dichloroquinoline					
12.5	11.55 ± 0.07	11.45 ± 0.04	11.41 ± 0.02	1.89	ND
Quinine					
60	11.65 ± 0.03	11.79 ± 0.01	6.52 ± 0.02	1.03	Observed
Untreated control	11.67 ± 0.05	11.83 ± 0.04	100% Mortality recorded	–	–

Treatment was initiated on day 8 post-infection. Drugs administered iv twice a day, for 3 days, within 12-h intervals and parasitaemia data before and 24 h post-treatment compared using paired Student t test (*p* < 0.05). Parasitaemia levels were similar in all the experimental groups before treatment (ANOVA, *p* >0.05)

*ND* not determined (100% mortality occurred)

At the two dosages used, 12.5 and 25 mg/kg iv, the trioxaquine showed a strong and promising antiplasmodial activity. At 12.5 mg/kg, parasitaemia decreased from 11.7 to 1.6 (86.6% clearance) and from 11.7 to 0.4 (96.4% clearance) at 25 mg/kg just after 24 h post-treatment and no parasite was observable under microscope at 48 h post-treatment. A potent anti-malarial activity was therefore exhibited by the artesunate–quinoline hybrid drug at the two dosages, resulting in a rapid parasite clearance. The treated animals also survived beyond 60 days post-treatment except for the 12.5 mg/kg, where death of one animal was recorded on day 4 post-treatment even after no parasite was observed under microscope.

Artesunate monotherapy at 12.5 mg/kg also showed a rapid parasitaemia clearance of 84.8% within the first 24 h post-treatment, and no parasite was observable under microscope at 48 h post-treatment. However, recrudescence was observed on day 8 post-treatment with all the animals succumbing to the infection by day 16 post-treatment (Fig. 3). Quinine at 60 mg/kg and 4,7-dichloroquinoline at 12.5 mg/kg did not show any significant parasitaemia reduction in the first 24 h post-treatment with all the animals in these experimental groups dying by day 12 and day 7 post-treatment, respectively (Fig. 3). Quinine, even after exhibiting a significant parasitaemia reduction in the 48th h post-treatment (44.9%), on further monitoring of parasitaemia showed progression of parasite growth for this group leading to death of mice (Fig. 3). Mice in untreated control group also succumbed to the infection by day 10 pi (Fig. 3).

Lack of recrudescence observed with the trioxaquine-treated animals could be attributed to the 4,7-dichloroquinoline, the partner drug to artesunate in the trioxaquine.

### Assessment of integrity of mouse blood–brain barrier

**Fig. 2:**
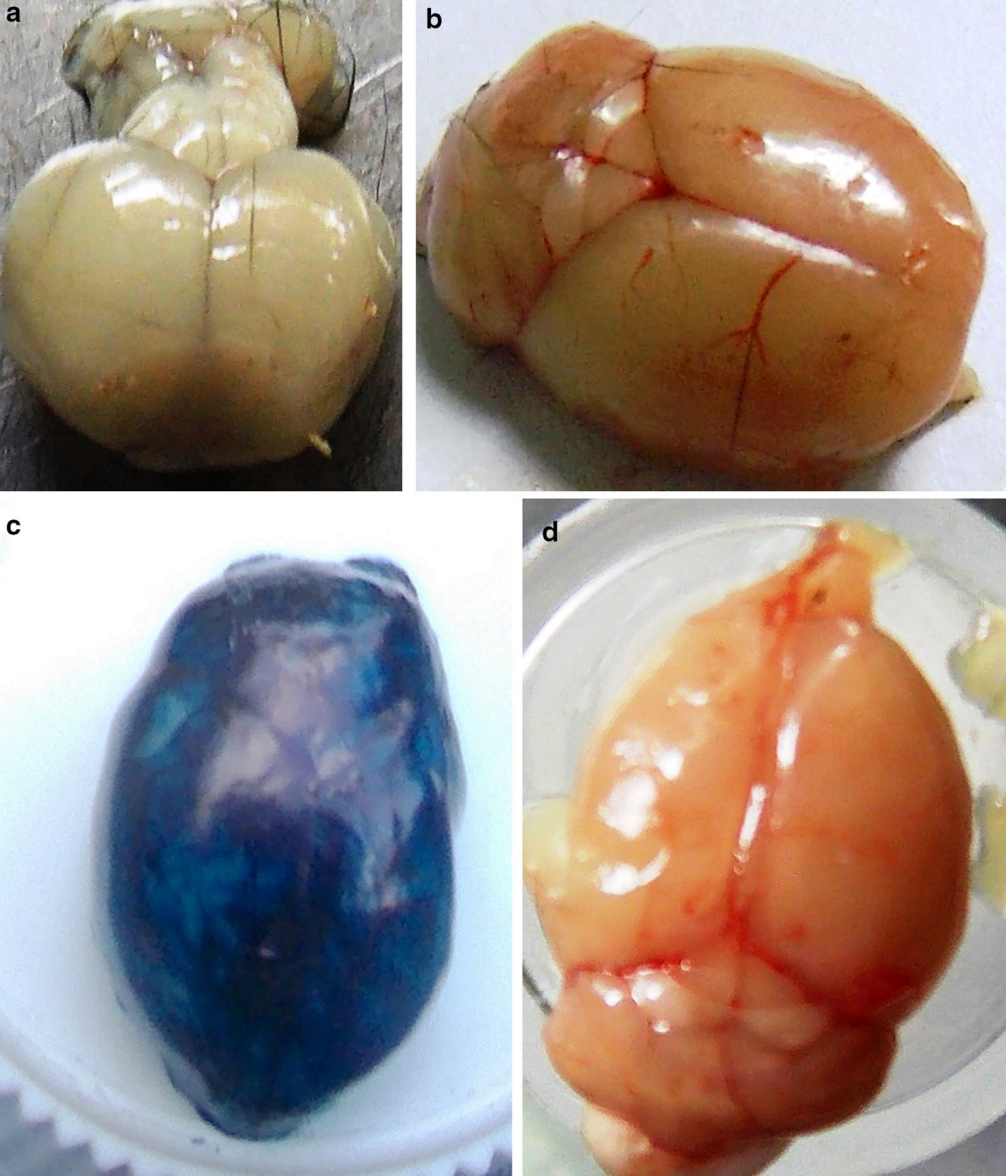
Evaluation of blood–brain barrier integrity with Evans Blue dye. The susceptible mice were first infected with *P. berghei* ANKA and the dye administered iv on day 8 pi after the infected mice had exhibited clear signs of ECM. The mice were euthanized 1 h later and the brain images documented. The same protocol was repeated on mice that were treated with trioxaquine at 60 days post-treatment. **a** Non-stained, *P. berghei* ANKA-infected C57BL/6 mouse brain on day 8 pi depicting hypoxia; **b** non-infected C57BL/6 mouse brain depicting normal blood supply; **c** brain staining for C57BL/6 mouse on day 8 pi; **d** depicts brain from the same animal model on day 60 post-treatment exhibiting recovery from experimental cerebral malaria, and lack of staining with Evans Blue dye

Damage to the blood–brain barrier (BBB) has been implicated and is believed to be one of the underlying mechanisms of pathophysiology of CM, as the leaky BBB would allow toxic or unwanted compounds to enter the brain, causing neurological dysfunction. Observations of the brain tissue in infected animals that were never injected with the 2% Evans Blue dye on day 8 pi showed a higher degree of brain whitening in C57BL/6 mice (Fig. 2a). The massive brain whitening observed in Fig. 2a is believed to be due to reduced or blocked blood flow at multiple sites in the brain (hypoxia), a confirmation of the phenomenon of pRBC sequestration in the brain microvasculature, which is one of the major hallmarks of cerebral malaria. Uninfected C57BL/6 mice (Fig. 2b) showed a normal healthy brain with normal blood supply, an absence of brain staining with Evans Blue dye indicating an intact BBB integrity. Observation of brains after the injection of the infected experimental animals with 2% Evans Blue dye on day 8 pi showed a higher extent of brain staining in the mice (Fig. 2c), a feature that substantiates the impairment of the integrity of the BBB. Observation of brain images of infected C57BL/6 mice at 60 days post-treatment exhibited complete recovery, return of normal blood supply and lack of brain staining by the Evans Blue dye (Fig. 2d), results that are comparable to Fig. 2b of a healthy uninfected animal.

### Evaluation of safety of trioxaquine in mice

Acute oral toxicity of trioxaquine was evaluated by using uninfected female mice that had been starved for 4 h, administered with oral dosages of 300 and 2000 mg/kg once. The test animals were then observed for 14 days post-dosing, basing the assessment on signs of severity, and/or death observed with test animals. All the animals did not show any sign of severity or toxicity as no changes in skin and fur, eyes and mucous membranes, as well as aberant respiratory activities were observed; also no tremors, convulsions, salivation, diarrhoea, sleep, and coma were observed. The mice survived past the 14 days of observation with 67% survival recorded with the dose of 2000 mg/kg. This is an indication that the exact lethal dose-50 (LD_50_) could be within 2000 mg/kg resulting to a possible therapeutic index (TI) value of >400, considering the oral lethal dose-50 to that of effective dose-50 (ED_50_) (i.e. LD_50_/ED_50_) ratio. The ED_50_ had previously been determined orally during in vivo antiplasmodial evaluation for the trioxaquine using the 4-day suppressive test [17]. This relatively weak toxicity indicates a very wide margin of safety for the test compound, implying that the compound could be a promising drug candidate for further exploration.

## Discussion

Infection with *P. falciparum* often has high chance of progressing into CM, a severe form of malaria that contributes to high malaria mortality in sub-Saharan Africa [9]. Children 5 years old and younger, expectant women at first trimester, and individuals with naive immunity visiting malaria-endemic areas are at increased risk of infection [5].

*Plasmodium falciparum* has shown a rising trend of evolution and emergence of resistance to the currently used drugs, even to the WHO gold standard anti-malarial, ACT [5, 10]. Also, very few anti-malarial drugs are in the clinical development pipeline. Optimizing use of existing drugs as well as use of rational drug development strategies, such as covalent bitherapy, could lead to enhanced, useful therapeutic lives of existing therapy as well as add new therapy to the existing anti-malarial drug repertoire [12, 13]. In covalent bitherapy, just as in combination therapy, the goal is to either delay or circumvent development of resistance. In trioxaquines, the fast-acting precursor (ART pharmacophore) provides rapid clearance of the bulk of parasite load while the quinoline moiety clears the remnant parasite that survives the effect of the former, until complete clearance is achieved [13]. This strategy has potential to improve the therapeutic effectiveness as well as delaying or circumventing the emergence of resistance to both individual precursors of the hybrid drug, besides overcoming the challenge of a long drug development pipeline for co-formulated ACT drugs [15]. The trioxaquine exhibited a higher efficacy compared to individual precursors alone (Table 1). Based on the curative test using established infection, the trioxaquine though exhibiting activities comparable to artesunate, proved to be more effective than both the parenteral artesunate, quinine and 4,7-dichloroquine (Table 1). The trioxaquine manifested a rapid parasite clearance of greater than 80% within 24 h post-treatment and no parasite was observable under microscope at the 48 h post-treatment for both dosages of 12.5 and 25 mg/kg (Table 1).

**Fig. 3:**
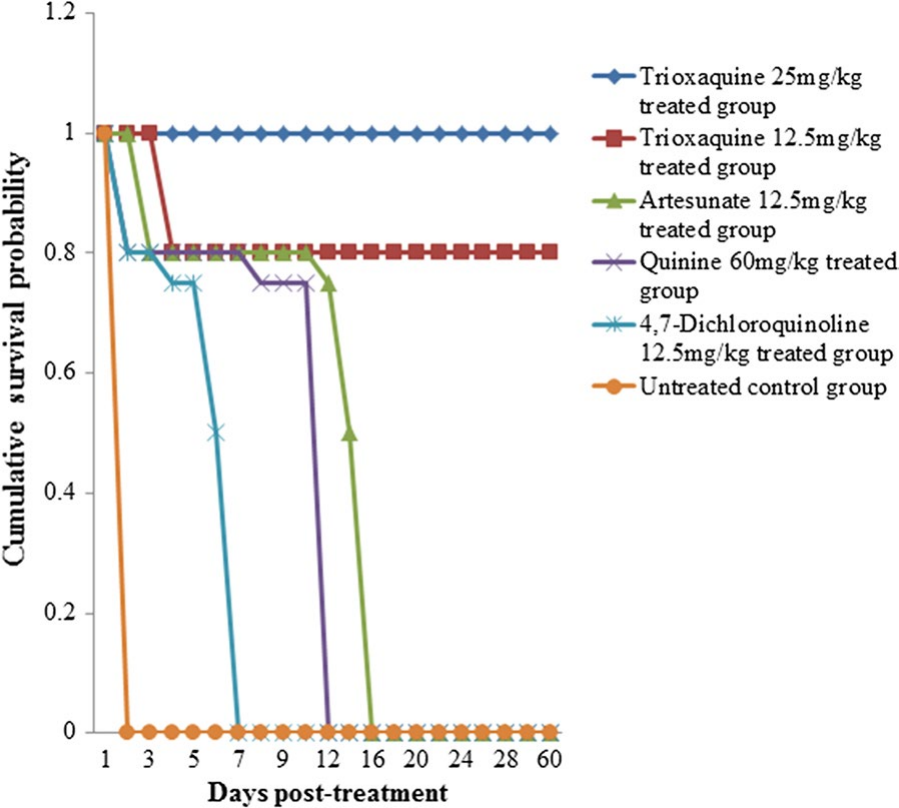
Survival probability analysis of the treated mice after infection with *Plasmodium berghei* ANKA, and treated on day 8 post-infection (pi) with various dosages of the trioxaquine, artesunate, 4,7-dichloroquine, and quinine. The mice were monitored for 60 days post-treatment. The experimental animals were first infected with an inoculum of 0.2 ml of approximately 1 × 10^5^ parasitized erythrocytes with *P. berghei* ANKA, treated with 25 and 12.5 mg/kg of the test drug on day 8 pi, twice a day for 3 days and their survival monitored for 60 days post-treatment. Quinine at 60 mg/kg, artesunate at 12.5 mg/kg, 4,7-dichloroquinoline at 12.5 mg/kg, and untreated control groups were included. Survival probability analysis using Kaplan–Meier software revealed that there was a significant reduction in mortality in trioxaquine-treated groups (*p* < 0.05) in comparison to the standards (quinine, artesunate, 4,7-dichloroquinoline). Apart from the trioxaquine treatment groups, all the other treatment groups, as well as the negative controls, had 100% mortality by day 16 pi

Long-term monitoring of animals treated with trioxaquine at both 12.5 and 25 mg/kg showed no recrudescence, with the animals surviving beyond 60 days post-treatment (Fig. 3). This is a clear indication of complete cure in the treated animals since no recrudescence was observed, an indicator that the dual drug could potentially circumvent or delay development of drug resistance.

Although rapid parasite suppression (84.8%) was observed in the first 24 h post-treatment with artesunate and no parasite was observable under microscope 48 h post-treatment, recrudescence was observed in this treatment group with all the animals succumbing to the infection by day 16 post-treatment. Even though the quinoline moiety, on the other hand, did not show any significant parasite reduction in the first 24 h post-treatment, its contribution in trioxaquine was manifested by lack of recrudescence in trioxaquine-treated animals even beyond 60 days post-treatment. The quinoline pharmachophore (4,7-dichloroquinoline) is believed to promote dual drug accumulation in the food vacuole, allowing the artemisinin-based partner (artesunate) to have a longer half-life [25], a possible explanation for the observed improved treatment outcome in the trioxaquine treatment groups compared to the artesunate-treated group. During treatment with 12.5 mg/kg of the trioxaquine, one mouse was lost on day 4 post-treatment. However, it was deemed that the death was not due to drug toxicity since even mice that received 25 mg/kg of trioxaquine had 100% survival. CM leads to a multi-organ dysfunction involving the liver, kidney and brain with the extent of the organs’ damage varying from individual to individual. Damage to these major organs are cited as the major reason behind CM deaths in infected individuals even after chemotherapeutic intervention [26]. This reason could support the loss of one animal in the 12.5-mg/kg trioxaquine treatment group even after parasitaemia monitoring had shown complete clearance. These results are in agreement with other previously related studies using the same experimental animals, which noted manifestation of neurological symptoms of CM between days 6 and 9 pi [18, 19, 27] and death in untreated animals by day 10 pi [28]. Recrudescence was also reported in artesunate-treated animals even at higher doses of 32 and 64 mg/kg, with all the treated animals succumbing to the infection and quinine being effective only at higher doses >120 mg/kg, which were not well tolerated by the experimental animals [29].

However, albeit in blood-stage infection, it was earlier reported that trioxaquines were capable of inhibiting parasites in mice and also affording curative effect in both the 4-day suppressive test and in established infection studies [13].

Two components have been implicated to be participating in the development of cerebral malaria, namely, the parasite-related factors and the host immune factors. Rupture of the hepatocytes leads to accumulation of the iRBCs within the brain microvasculature as the parasite invades the blood stream [30]. On the blood side of the BBB, *P. falciparum*-infected RBCs (*Pf*RBCs) cytoadhere to the brain endothelium by binding of *P. falciparum* erythrocyte membrane protein 1 (*Pf*EMP1), a specific cell-surface ligand expressed by iRBCs [31]. The sequestration of iRBCs triggers activation of the endothelial cells (ECs), which in turn lead to inflammatory responses where there is release of pro-inflammatory cytokine tumour necrosis factor (TNF) [32]. This cytokine is known to upregulate endothelial receptor cells, resulting in upregulation of intracellular adhesion molecules 1 (ICAM-1) and vascular cellular adhesion molecules-1 (VCAM–1) [33]. All these factors would eventually lead to numerous downstream vascular effects, such as increased vasoconstriction, reduced cerebral blood flow to several sites in the brain, vascular obstruction, oxygen starvation, and even disruption of the integrity of the BBB [34]. The observed whitening of the susceptible strain brains (Fig. 2a) could therefore be attributed to the reduced or the complete blockage of cerebral blood flow to several sites in the brain, a condition termed as hypoxia.

The BBB is comprised of specialized endothelial cells (ECs) that function as a selective permeable membrane to control nutrients and ion transport into the brain and to bar unwanted molecules or compounds into the brain [35, 36]. The ECs are sealed together by the presence of tight junctions. However, loss of tight junctions has been observed in areas of iRBC sequestration, leading to creation of openings or gaps within the BBB [36]. Such gaps or openings could function as a gateway for the entry of toxic molecules or compounds into the brain, thus a pointer to the dysfunctioning BBB, which is a major pathological event associated with CM [34, 37]. This was well demonstrated in this work through staining of the human cerebral malaria (HCM) mouse models’ brains with the 2% Evans Blue dye (Fig. 2c), an indicator of the impaired BBB integrity in the infected mice. The data of this study are similar to other previously related studies that confirmed impairment of the integrity of BBB in susceptible animal models and subsequent brain staining with Evans Blue dye [27, 38–40].

Good safety profile was exhibited as the drug was well tolerated in mice even at high dose of 2000 mg/kg administered in an acute oral toxicity test, with 67% survival. The remarkable mice survival indicates that the exact LD_50_ could be within 2000 mg/kg. However, it should be noted that this is a dosage 40 times the curative dose of 25 mg/kg. Considering the possible LD_50_/ED_50_ ratio, a possible TI value >400 reflects a wider margin of safety for the trioxaquine. The same safety profile was manifested in in vitro cytotoxicity studies where the selectivity index (SI) of the trioxaquine was determined by comparing the IC_50s_ of Hep2 cell line and that of the CQ-resistant parasite (W2) for the trioxaquine [17]. The high SI value obtained (>2762) indicates that the high antiplasmodial activity for the trioxaquine observed during in vitro antiplasmodial evaluation was due to its activity and not due to its cytotoxicity [17]. A similar class of trioxaquine was reported to have antiplasmodial activity against all the erythrocytic forms of the parasite, including the gametocytes with TI value range of 23–100 considered safe [13]. The high safety profile exhibited both in vivo and in vitro by the trioxaquine would therefore be attributed to the fact that the individual precursors for the trioxaquine are already indicated for clinical use in the management of malaria.

The remarkable efficacy and good safety profile of the trioxaquine observed imply that if the findings could be replicated in clinical studies, then the drug has potential for use in management of CM.

## Conclusion

The remarkable in vivo results for the trioxaquine obtained by the established infection test, as well as the post-treatment survival data, clearly indicate that covalent bitherapy is a viable strategy for development of future anti-malarial agents for management of CM. An encouraging safety profile of the drug in mice with a therapeutic index (TI) value of >400 is not surprising since the precursors are part of drugs already indicated for clinical use. Overall therefore, the curative effect together with good safety profiles observed with the trioxaquine clearly demonstrate its potential as a drug candidate for management of CM, especially in a time of shrinking anti-malarial armamentarium for management of CM.

## Abbreviations

*Pf*iRBCS: *Plasmodium falciparum* infected red blood cells
NACOSTI: National Commission for Science Technology and Innovation
BBB: Blood brain barrier
CBRD: Centre for Biotechnology Research and Development
CTMDR: Centre for Traditional Medicine and Drug Research
ECs: Endothelial cells
ED_50_: Effective dose causing clearance of 50% of the parasite
ICAM-1: Intracellular adhesion molecule 1
iRBCs: Infected red blood cells
CM: Cerebral malaria
LD_50_: Lethal dose causing death of 50% of test animals
TI: Therapeutic index
TNF: Tumour necrosis factor
SI: Selective index
SERU: Scientific Ethics Review Unit
WHO: World Health Organization
VCAM-1: Vascular cellular adhesion molecule 1
